# Halting and re-issuing of the Zambia community health strategy (2017–2021): a retrospective analysis of the policy process and implications for community health systems

**DOI:** 10.1186/s12913-024-11419-9

**Published:** 2024-08-22

**Authors:** Malizgani Paul Chavula, Adam Silumbwe, Margarate Nzala Munakampe, Joseph Mumba Zulu, Wanga Zulu, Charles Michelo, Chama Mulubwa

**Affiliations:** 1 Department of Community and Family Medicine, School of Public Health, University of Zambia, Lusaka, Zambia; 2 Department of Epidemiology and Global Health, Umeå University, Umeå, Umeå, 901 87 Sweden; 3 Department of Health Policy and Management, School of Public Health, University of Zambia, P.O Box 50110, Lusaka, Cell, +260976085894 Zambia; 4 Department of Public Health, National TB and Leprosy programme, Ministry of Health, Lusaka, Zambia; 5 Strategic Centre for Health Systems Metrics (SCHEME), Global Health Institute, Nkwazi Research University, PO Box 50650, Lusaka, Zambia

**Keywords:** Community, Community health, Community health system, Community health policy, Strategy, Policy analysis, Zambia

## Abstract

**Background:**

Over the years, low-and middle-income countries have adopted several policy initiatives to strengthen community health systems as means to attain Universal Health Coverage (UHC). In this regard, Zambia passed a Community Health Strategy in 2017 that was later halted in 2019. This paper explores the processes that led to the halting and re-issuing of this strategy with the view of drawing lessons to inform the development of such strategies in Zambia and other similar settings.

**Methods:**

We employed a qualitative case study comprising 20 semi-structured interviews with key stakeholders who had participated in either the development, halting, or re-issuing of the two strategies, respectively. These stakeholders represented the Ministry of Health, cooperating partners and other non-government organizations. Inductive thematic analysis approach was used for analysis.

**Results:**

The major reasons for halting and re-issuing the community health strategy included the need to realign it with the national development framework such as the 7th National Development Plan, lack of policy ownership, political influence, and the need to streamline the coordination of community health interventions. The policy process inadequately addressed the key tenets of community health systems such as complexity, adaptation, resilience and engagement of community actors resulting in shortcomings in the policy content. Furthermore, the short implementation period, lack of dedicated staff, and inadequate engagement of stakeholders from other sectors threatened the sustainability of the re-issued strategy.

**Conclusion:**

This study underscores the complexity of community health systems and highlights the challenges these complexities pose to health policymaking efforts. Countries that embark on health policymaking for community health systems must reflect on issues such as persistent fragmentation, which threaten the policy development process. It is crucial to ensure that these complexities are considered within similar policy engagement processes.

## Background

Global efforts to attain Universal Health Coverage (UHC) have emphasized the strengthening of community health systems (CHSs), as they are at the core of addressing local public health problems [1]. This call has resulted in various country level investments in policies or strategies to govern the development and evolution of CHSs [2]. According to Schneider et al., 2016, CHSs consist of a set of local actors, relationships and processes, engaged in producing, advocating for, and supporting health in communities and households outside of, but existing in relationship to, formal health structures [3]. CHSs represent a subset of the formal health system and exhibit tenets such as complexity, adaptation, emergence and self-organizing [4]. Further, they form a critical foundation for achieving positive health outcomes in many countries, with various community actors within CHSs providing diverse healthcare services such as comprehensive home-based care, family planning, counselling, advocacy, legal support, and referrals [5, 6].

The governance of CHSs requires national health policies and strategies that provide an enabling environment for the design and management of health programmes at the community level. The challenge, however, is how to develop health policies or strategies that nurture and strengthen the CHS’ capacity to respond to implementation realities that influence the scale and sustainability of community programmes [7]. According to George et al., 2016, developing such health policies or strategies requires the humility to understand communities as social systems with several elements that interact with health policies in complex ways [8]. Like formal health systems, CHSs are the intersection point for cross-sectorial efforts at the community level and are therefore impacted by dynamics external to the health sector [9]. Understanding CHSs therefore entails the recognition that ‘top-down’ policies and programmes always exist in tension with ‘bottom-up’ realities. Communities are not programmable; interventions must be adapted to local contexts, with meaningful community engagement that acknowledges power relations both within communities and in formal health systems [4, 10]. Further, it is important to note that most health policies targeting CHSs tend to only focus on Community Health Workers (CHWs), although health gains at the community level involve a far greater array of community and health system factors beyond these cadres [11]. Schneider and Lehmann, 2016 argue for the need to holistically focus on systems when designing policies and strategies for community health [3].

Over the years, many low-and middle-income countries (LMICs) have adopted several policy initiatives to strengthen CHSs to attain UHC. In Zambia, initial community health policy efforts were primarily concentrated on the CHWs [12]. For instance, CHWs were central to the National Health Strategic Plan 2006–2010 which emphasized community-based health service delivery. In 2010, Zambia developed the National Community Health Worker and the Community Health Assistant Strategies, which aimed to reposition and expand the available cadres of frontline health workers at the community level [13, 14]. To address the broader CHS beyond just the CHWs, Zambia passed the first Community Health Strategy 2017–2021 [15]. The goal of this strategy was to contribute towards improved health service delivery and attainment of national health priorities and goals, through strengthened provision of preventive, promotive and selected curative services at community level.

However, this strategy was halted in 2019, shortly after implementation began, and a new one was re-ssued [16]. It remains unclear as to why the decision was made to halt the 2017 CHS Strategy a little over a year of implementation. This paper explores the processes that led to the halting and re-issuing of the Community Health Strategy document with the view of drawing lessons to inform the development of such strategies in Zambia and other similar settings.

## Methodology

### Study design

We used a qualitative case study design to explore stakeholder perspectives regarding factors that triggered the development of the current Zambian National Community Health Strategy 2019–2021 within a year of implementing the 2017–2021 strategy. A case study is described as an in-depth exploration, from multiple perspectives, of the complexity and uniqueness of a particular policy, institution, program, or system in a ‘real-life’ context” [17]. Case studies are appropriate for exploring the ‘why’ and ‘how’ questions in health policy change studies [18, 19]. In this study, we adopted the halting and re-issuing of the Zambian community health strategy between 2017 and 2019 as our case. Specifically, we focused on stakeholders who had either participated in the development, halting, and re-issuing of the two strategies respectively.

### Study participants and data collection

The data collection took place in March 2020. We collaborated with the Departments of Community Health, and Health Promotion and Social Determinants of Health at the Ministry of Health (MoH) to identify participants that were involved in the development of the first and second Community Health Strategies. We contacted 25 key informants to schedule interviews via phone calls and emails, using information from the attendance registers of the policy stakeholder meetings obtained from the two departments. Of the 25 participants, 20 accepted our invitation to participate in the study and were interviewed. The participants included stakeholders from the MoH, cooperating partners and other non-government organizations.

All the interviews were conducted in English and in-person within the workplace of the participants by experienced qualitative data collectors. Each interview lasted approximately 35–60 min. We collected data using a semi-structured interview guide that comprised of broad questions and probes on contextual factors that influenced implementation, content, design, cancellation of the 2017 strategy as well as development or re-issuing of the 2019 Strategy. We conducted interviews until no new data emerged in line with the principle of theoretical saturation. Additionally, we piloted and refined the interview guides based on feedback from five civil society stakeholders.

### Data analysis

All audio recordings were transcribed verbatim and imported to NVivo 12 Pro Software for coding and analysis. We analysed the data using thematic analysis as described by Braun and Clarke [20]. We developed a coding structure based on initial reading of small sample of six transcripts. All the co-authors extensively discussed the preliminary codes and later grouped them according to main and sub-themes. After agreeing to the coding structure, the four co-authors (AS, MPC, CM, MM) separately coded the rest of the 14 transcripts. During the analysis, the research team regularly met to discuss emerging codes, update the coding structure and check for inter-coder reliability [21].

### Ethical considerations

We sought ethical approval to conduct this study from the Excellence in Research Ethics and Science Converge (ERES) Ethics Committee, Reference No.2019-May-033. We further obtained permission from the National Health Research Authority and the MoH. We sought informed consent from all the participants before the interviews. Participation was voluntary and non-remunerable. Prior to commencement of the interviews, all participants were assured of confidentiality. This study was performed in accordance with the Declaration of Helsinki [22].

## Results

The results are organised according to three main themes. First, we describe the context and rationale of the Community Health Strategy 2017–2021 and explain the reasons for halting of this strategy within a year of enactment. Secondly, we describe the process and the politics during the development of the 2019 strategy. Thirdly, we discuss the implications of the 2019 strategy development processes on implementation and the CHS development.

### Context and rationale for halting the Zambian Community Health Strategy 2017–2021

In 2017, Zambia passed the first Community Health Strategy whose main objective was to empower communities to take responsibility for improving their health through community-led interventions in line with principles of primary healthcare. Specifically, the strategy sought to revitalize community health structures as well as define the mandates that shape relationships within, and between community and formal health systems structures. The MoH Department of Health Promotion and Social Determinants spearheaded the development of 2017 strategy in collaboration with an international consultant while the European Union and the World Bank funded all activities.

*“I may not mention all the steps*,* but I remember that this was a very consultative process. Every couple of months*,* we would get an invitation from the Health Promotion Department to say there is this part of the strategy that needs to be discussed*,* can we sit down together as partners? I think even just at the beginning of the process there were like needs assessments meetings where partners would be called to indicate what the issues where when it comes to community health.” [K11*,* 17*,* NGO Staff].*

### Reasons for halting and revision of the 2017 CHS strategy

#### Limited engagement of stakeholders

The key informants who did not participate in the development of the first strategy explained that although the 2017 strategy had set ambitious targets, it was not a good document as it had several limitations that led the MoH to halt it in little over a year of implementation. For example, some of the challenges included limited engagement of stakeholders and lack of clear details on how the policy was to be implemented. One participant expressed the various concerns with the 2017 strategy and how they led to halting it:

*“After the first strategy was done*,* it was realized that it still missed some of the critical issues that needed to be addressed at community level. The strategy seemed to have focused more on the structural aspect as opposed to the community. A team of technocrats was then put together to relook at the strategy and begin the process of halting it to address some of the inadequacies.” [K11*,* 3*,* Government Staff].*

#### Misalignment with national frameworks

One of the important factors that led to the halting of the 2017 strategy was the need to align it with the national development framework. Immediately after the Community Health Strategy 2017–2021 strategy was passed, the country adopted a new development framework through the 7th National Development Plan 2017–2021 (7NDP) and the National Health Strategic Plan 2017–2021 (NHSP). The process of developing the 7th National Development Plan 2017–2021 (7NDP) started almost at the same time as that of 2017 Strategy. Upon close examination, it was discovered that the 2017 strategy did not adequately align with these two national frameworks. The lack of aligment meant that the Strategy lacked supportive implementation environment. Further, this situation made both implementation of the 2017 strategy and sourcing for funding difficult, as one stakeholder explained.

*“It firstly started with the Community Health Unit*,* we had difficulties in utilizing the strategic document*,* and yes*,* we had problems utilizing the strategic document. As a unit*,* we had failed. Looking at the health priorities that are in the NHSP they did not fit so it was very difficult for us to implement certain activities. It was very difficult for us to unlock certain funding*,*” [K11 14*,* Government Staff.*

In addition, the key informants explained that the 2017 strategy had to be revised to incorporate key aspects of the NHSP. These included embracing a multi-sectoral approach to planning and provision of health services at all levels and bringing healthcare closer to people in the most affordable manner. Another informant narrated.

*“We analysed the document [referring to the 2017 strategy] to say no it’s not a good document*,* it needed some refinement*,* and it needed to be aligned to the existing national development framework. That is how the whole idea of revising the community health strategy was birthed [2*,* KII*,* Government Staff].*

To further explain why this strategy was halted, one informant shared her views on what a good policy should consist of, which according to her seemed to be lacking in the 2017 strategy.

*“It’s a strategic document where planned activities that may be implemented are documented with the sole purpose of providing healthcare at the community level. It is a document where some of these things are well spelt out and it also has an investment case.” [KII*,* 2*,* Government Staff].*

#### Lack of policy ownership

The key informants narrated that policy implementors shared a general feeling of lack of ownership of the 2017 strategy. The Department of Health Promotion and Social Determinants that spearheaded the development of this strategy had been restructured, giving birth to the Community Health Unit (CHU). This restructuring and splitting of the department seemed to have created a vacuum in terms of policy ownership. According to the key informants, the new CHU had to develop a strategic document that they could preside over and align with their new mandate. Lack of ownership was also worsened by the engagement of an international consultant during the development of the first strategy. Some key informants who did not participate in the development of the 2017 strategy felt that the consultant lacked a full understanding of the Zambian context and CHS, failing to capture and reflect all stakeholder perspectives as one key informant expressed.

***“****Because that strategy didn’t really have any ownership within the Ministry of Health. It was sought of siting with Department of Health Promotion and Social Determinants at the time it was developed. Once there was the Community Health Unit*,* I think people were quick to realize that this strategy wasn’t really fit for people in the community health unit” [ KII2*,* MoH Staff].*

Furthermore, some key informants reflected on and compared the leadership provided during the development of the 2017 strategy to the re-issuing of the 2019 strategy. They indicated that changes in leadership at the MoH after the development of the 2017 strategy may have contributed to its failure. For example, they stated that when a particular leader was transferred to a different department or government ministry, it left a leadership vacuum, which often affected the continuity of agendas as aptly put by a key informant.

*“Within the directorate of Public Health*,* the Department of Health Promotion was split with the Community Health Unit. During this*,* time the director in health promotion who had participated in the development of the first strategy had already moved and that is when the problem started. This also may have affected how other members of the department explained the intention and aspiration of the first strategy.” [KII*,* 7*,* Government Staff].*

#### Persistent fragmentation and coordination challenges of community health interventions

According to the key informants, another major reason for the revision of the 2017 strategy was the inadequate response to the fragmentation of community health services. They explained that community volunteers continued to be trained and remunerated differently across programmes. Similarly, there was no standardised procedure for implementing of the various community programmes. This negatively affected service provision at community level, as volunteers prioritized paid programmes over unpaid ones. In addition, the 2017 strategy did not address fragmentation in the funding of community-based health interventions that were driven by international partners. It was unclear how partners were required to engage with the communities and coordinate in areas with multiple partners. One key informant recounted.

*“I think the major thing is that there were inadequate guiding principles with the partners on how they should engage with the community. Each partner came with their own way of doing things. There was no clarity about harmonizing of volunteers; harmonization of payment …. that is where the birth of the revised strategy come from*,* to ensure that we guide all partners on how to go about implementing interventions in the community.” [K11*,* 13*,* NGO Staff].*

Additionally, the strategy had to be revised to provide for the cordination of community organizations. In some cases, the MoH was unaware of certain organizations providing services in the community, and only discovered them when a problem arose. Thus, the halting of the 2017 strategy sought to provide a better framework for the coordination of partners working within the CHS space by streamlining partner interest with community priorities.

*“Then the other challenge is partner co-ordination; you know there are a lot of partners that are implementing community activities. Sometimes we do not even know the partners; we tend to know about them when there is a problem.” [K11*,* 5*,* Government Staff]*.

### Re-issuing of the Community Health Strategy 2019–2021

Despite the shortcomings of the 2017 strategy, all the key informants emphasized the necessity and significance of implementing a functional and well-aligned strategy. This led to the re-issuance of the Community Health Strategy 2019–2021. In the following section we outline some of the factors that facilitated the re-issuance of this strategy.

#### The role of political actors in driving the process

The key informants narrated that the revision of the 2017 strategy was championed by the then Minister of Health in 2018 who not only identified community health as the backbone of public health services but also as a critical driver toward the attainment of UHC. The Minister thus provided both technical and political will from the government to facilitate the halting and re-issuing of the strategy with the view to strengthen CHS in Zambia. A key informant had this to say.

*The technical support more especially from our Minister*,* you know our Minister is health inclined. He realized that community health is the backbone for of all health services. We got really a lot of support*,* he put us at speed to ensure that the document was finalized in good time and knowing we only had limited time. I am sure; due to his influence*,* people have attached seriousness to the document. [K115*,* Government]”*.

*“I think the most important thing that led to the development of this new strategy is the political will. I think political will facilitated the process of developing and ensuring that there is a revised and updated community health strategy.” [KII*,* 2*,* Government Staff]*.

#### Stakeholder engagement during the community health strategy 2019–2021 development process

Similar to the 2017 strategy, several stakeholders reported playing different roles during the development of the Community Health Strategy 2019–2021. Most of the key informants who participated felt that there was adequate engagement of stakeholders during the situational analysis, consultative meetings, reviewing and launching of the strategy. One key informant described:

*“So*,* what we did as a unit*,* we had to bring together partners. We had to start with a situation analysis*,* that was the first step we did. We looked at what was prevailing on the ground and felt there was a need to revise the strategy. Then the second step*,* we had consultative meetings with various stakeholders” [K1I 11*,* Government Staff]*.

While some partners were responsible for financing the process, others provided technical support by shaping and refining the content of the re-issued strategy to align with the aspirations of the government in the 7NDP and NHSP. Specifically, some key informants working under the MoH reported holding consultative meetings with various stakeholders in community health. Interviews were also conducted with people at different levels of government, including political figures and healthcare providers.

*“Some partners*,* of course*,* some supported financially*,* some provided us with technical support during the development of the strategy. For example*,* Amref Health Africa did provide financial support and technical support*,* and then there was CIDRZ*,* JHPIEGO and JICA which that provided technical support. I think most of them its technical support*,* but Amref Health Africa and the Ministry of Health; I think provided financial support also came in. [KII*,* 14*,* Government Staff].*

The key informants representing international partners classified the policy process as consultative. They participated in the meetings and drafting of the strategy and its content as one of them recounted.

*“The development of this strategy was participatory by all partners. We had supporting partner meetings*,* and after that*,* the draft was sent to all participants. We put our input*,* and we sent it back*,* and when it come back to us*,* we had another workshop where we had to refine it. Another meeting was called to look at how we harmonize some thematic areas*,* looking at how all strategic documents run concurrently with the ministry of health plans.” [KII*,*10*,* NGO Staff].*

However, other key informants, particularly the community representatives reported that their involvement was limited to attending MoH-driven meetings, which did not provide a platform to adequately inputs into the document.

*“I think we were part of the meetings to develop the strategy. We were invited for several meetings. However*,* this process seemed to have been mostly for other professional health workers. I do not think I remember anytime that we were asked to add specific issues to the document during these meetings.” [K1I*,* 16*,* Community Health Worker].*

The key informants who were only involved in the development of the 2017 strategy were displeased with the stakeholder engagement during its revision. They felt that this process prioritized the involvement of funding organizations at the expense of community actors and some departments in the MoH, including the Department of Health Promotion and Social Determinants of Health that spearhead the development of the 2017 strategy. The failure to consult such departments was attributed to a lack of consensus among respective department leaders regarding the content of the re-issued strategy. There was also fear that some individuals involved in developing the 2017 strategy would opt to retain the initial content by resisting certain amendments. Furthermore, the Community Health Unit, which led the 2019 strategy, faced challenges in bringing together various stakeholders because it was recently formed and had not yet established relationships with many of them. A key informant from one of the departments that was not invited stated.

*“However*,* other stakeholders were not invited… I think we also have the agriculture extension officers these are also found in the community yes*,* traditional leaders and chiefs*,* ward councillors public health specialists*,* nurses” [KII 14*,* Government Staff].*

### Implications of the community health strategy 2019–2021 on CHS development in Zambia

#### Short implementation period affecting programmatic learning and evaluation

The key informants narrated that although the 2019 strategy had addressed gaps identified in the 2017 strategy, there was insufficient time left to implement it to achieve the intended policy outcomes. They complained that the short implementation period (2019–2021) limited the acquisition of sufficient evidence and lessons to inform the development of a more integrated CHS strategy. Further, most key informants noted that this limited timeframe could hinder evaluating the effectiveness of the strategy in addressing key challenges to the CHS such as selection, training, and supervision of CHWs. Moreover, there was also inadequate time to effectively communicate the policy changes to the frontline health providers and community actors responsible for implementing the revised strategy as one key informant lamented.

“*Some of these strategic documents are usually planned for 5 years*,* 2017–2021*,* but with this re-issued strategy*,* it is 2020–2021. So*,* you are looking at the time constraint of which to implement these activities is too short. For example*,* most of these other policies within health have started reviewing their activities*,* whilst this is just starting. Honestly*,* if you look at the community health strategy*,* we do not really have time. You know after a document like that has been done*,* you need time to implement the activities that you have spelt out.” [KII*,* 8*,* Government Staff].*

#### Lack of dedicated human resources to implement the strategy

Some key informants felt that even with the 2019 strategy, the Community Health Unit still lacked dedicated staff at the provincial, district, and health facility levels to implement all activities in the re-issued strategy. They questioned the lack of clarity concerning the roles of healthcare staff beyond CHWs. Further, they felt that there was a need to clarify how this new strategy would be implemented and map out related frontline staff and volunteers. This, they said, would address potential implementation gaps and requisite skills for provision of community health services.

*“I think one of the things that is strange about this strategy is that we don’t even know how much it may require to implement it. So*,* because once you have the cost implementation it allows to engage in different discussions on what kind of human resources do we need. Remember also that Community Health Unit does not have structure across the various governance levels. Then also*,* what kind of human resources are we to rely on? With what kind of competencies if we are to achieve some of the strategic targets.” [K11 4*,* NGO Staff].*

#### Engagement of other CHS stakeholders in other sectors

There was a general acknowledgement among the key informants on the need to engage all community health stakeholders in various capacities, particularly about funding and technical support during the re-issued strategy’s implementation period. Those stakeholders with established community structures such as the Ministry of Community Development and Social Welfare, the Ministry of Agriculture (MoA) and the Ministry of Chiefs and Traditional Affairs were considered essential to the success of this CHS policy. Some of them had been left out during the development of the re-issued strategy. Consequently, this could lead to more time spent on training, lobbying and creating buy-in, a challenging task given the limited implementation timeframe of the re-issued strategy. Two key informant informants explained:

*“The Ministry of Health should lead the implementation of the strategy*,* but we also have other players. Apart from them*,* we have other donors that support the health sector in Zambia; we have implementing partners*, i.e.,* local*,* and international NGOs*,* faith-based organizations and then also the community itself; the community structures*,* the churches at community level you know*,* traditional leaders as well… they can’t be left out. So those are some of the stakeholders that should be involved.” [KII6*,* MoH]*.

*“I think I also mentioned like from Ministry of Agriculture we have the agriculture extension officers. Those in some areas help a lot in nutrition activities. In addition*,* we also have the chiefs*,* those from the Ministry of Traditional Affairs. I know it cannot apply in urban areas but in the communities. You know Zambia has got a lot of rural parts*,* so I think in the rural areas*,* those are key. The people from Ministry of Chiefs and Traditional Affairs should be play a role if this strategy must succeed. [KII 17*,* Government Staff].*

## Discussion

This paper explored the process that led to the halting and re-issuing of the Community Health Strategy in Zambia. Several factors contributed to halting the 2017 strategy and re-issuing the 2019 strategy, including the need to align this strategy with existing broader policies like the National Development Plans and the influence from the highest office in the health sector at the time. However, inadequate stakeholder engagement in both the 2017 and 2019 strategies, lack of consultation with the community, changes in actor involvement during revision of the 2017 strategy and revising this strategy a little over a year after its implementation hindered the meaningful implementation and evaluation of the re-issued 2019 strategy. Additionally, this policy process inadequately considered the key tenets of community health systems. In the following, we discuss the halting and re-issuing of the CHS strategy and reflect on how these events may affect community health systems in Zambia.

The halting of the 2017 strategy to realign it with the strategic national development framework such as the 7th National Development Plan which had come into effect slightly after this strategy, underscored the pivotal role assigned to community health within the broader national development agenda [23]. Additionally, the establishment of a separate Community Health Unit within the MoH highlights the recognition of the importance of CHSs. However, the short time of existence of this unit may have made it challenging to galvanize all policy stakeholders and garner the required consensus to revise and successfully deploy the re-issued 2019 strategy. This unit reportedly operated only at the national level, where it was not fully functional and lacked structures at lower levels of the health system. This implies that implementing the 2019 CHS strategy may be a challenge due to the lack of supportive organizational structures, including personnel. These findings underscore the importance of not only situating CHS development within the national context but also establishing appropriate supportive structures such as personnel and finance, to facilitate strategy implementation and the attainment of goals [16, 24].

The limited involvement of community actors raises doubts about whether the halting of the 2017 strategy and the re-issuing of the 2019 strategy fully embraced the complex-adaptive nature of CHSs. Communities are seldom considered in most bureaucratic, top-down approaches to community health policy engagement [25]. Top-down health policy processes often fail to address the problem of accessibility of health services for underserved communities [26–28]. In this study, we found indications of a predominantly top-down policy revision process. Although CHWs were invited to the consultative meetings, they were not given a platform to have their views incorporated in the 2019 strategy. Recognizing and ensuring that health policy processes respond to local realities and contexts, while acknowledging the multi-layered dynamics of power, is crucial in building resilient CHSs [29, 30]. The inclusion of CHWs in consultative meetings with more powerful stakeholders may have hindered their ability to participate and contribute effectively to the content of the 2019 strategy. Community actors must have their perspectives captured if the policy process has to effectively address CHS issues [8]. Moreover, strengthening CHS involves leveraging existing formal and informal networks within communities, including CHWs, religious, traditional, government and private sector leaders, to bridge gaps in community health services [30]. Policy development processes ought to embrace strategies that enable CHS actors to mobilize, collaborate, and collectively act on health issues [8, 31].

Furthermore, involving various actors builds trust and legitimizes the policy process, as CHS are complex adaptive systems with multiple interactions and feedback mechanisms [4, 32, 33]. In the Zambian context, these may include stakeholders beyond the health sector whose decisions shape community health and well-being such as Ministries of Community Development and Social Welfare, Agriculture and Chiefs and Traditional Affairs who are key to the CHS but were left out during the re-issuing of the 2019 strategy. Although most stakeholders described the revised 2019 strategy as having been consultative and inclusive, a key department within the MoH that spearheaded the earlier 2017 strategy did not participate. This selective involvement, often determined by authority figures, may have excluded relevant or controversial actors, affecting the development of a comprehensive 2019 strategy that could effectively address challenges in CHSs, such as fragmentation and coordination of interventions. Moreover, such exclusions can undermine the re-issued 2019 strategy ownership, reduce buy-in, and strain relationships among policymakers, negatively impacting its implementation and stewardship. Addressing these issues demands a policy process that negotiates disagreements, communication and leadership challenges among key actors [34, 35]. Promoting inclusiveness, transparency, and accountability among policy actors positively impacts health systems change [36, 37]. Indeed, policies implemented without adequate stakeholder participation may fail to gain legitimacy or retain their original intent during implementation [38].

Most of the key informants acknowledged the significant influence of the leadership and political power, exemplified by the then Minister of Health’s drive to expeditiously halt the 2017 strategy and re-issue the 2019 strategy. Cultivating political will, particularly from political leadership, can greatly facilitate health policy processes. Understanding the role of such key policy actors and how they wield power may be crucial for aligning policy content with the needs of the CHS [39, 40]. However, negotiating policy content with higher-level officials presents a formidable challenge for lower rank officials who are mostly the ones sent to represent their departments during the policy consultative meetings. Additionally, support from certain leaders has the potential to compromise the independence of actors in negotiating policy positions for fear of going against higher authorities’ interests and agenda. Interestingly, none of the stakeholders in this study highlighted these power differentials and their potential negative effects on the policy process.

Lastly, the major challenge with both strategies was the limited time available to implement and learn from both. The participants expressed concerns about the need for sufficient time for actors to glean insights for effective programing, amid existing weak monitoring and evaluation systems of CHW programs and other community health activities. This lack of learning time could impede the gathering of critical evidence to inform the development of future comprehensive CHS strategies that effectively contribute to attainment of UHC in Zambia and similar LMIC settings [41].

### Strengths and limitations

To enhance the credibility of our findings several key actors in both CHS strategies (2017 and 2019) were interviewed to get their perspectives on the policy process [42]. This facilitated triangulation of perspectives among participants, enabling us to distinguish between mere opinions and actual occurrences during this policy process. Further, the team comprised of seasoned senior qualitative researchers and experienced policy analysts that oversaw the data collection and analysis process. Four team members conducted the data analysis, which ensured an iterative process of developing and modifying the identified thematic areas. The use of the case study also provided a platform to explore the policy process and context in detail. A major issue with a retrospective policy analysis is the recall bias, in that people views on 2017 strategy are bound to be forgotten after the lapse in time. However, we also believe that this study draws lessons that could potentially shape the development of such CHS strategies in Zambia and other similar settings. Lastly, the implementation of the halted strategy was relatively brief, posing a challenge to comprehensively document efforts and practices contributing to governance issues within the policy.

## Conclusion

The study reflects on the intricate nature of CHS, highlighting the challenge of capturing this complexity in health policy development efforts. Nevertheless, it’s evident that policies addressing CHS are influenced by global and national agendas, as well as institutional power dynamics within the policy process. Countries engaging in health policymaking for CHS must consider these complexities including ongoing fragmentation and ensure their incorporation into policy agenda setting and engagement processes.

Several reasons were cited for halting and re-issuing the community health strategy in Zambia. However, whether the re-issued CHS policy effectively addresses the fundamental principles of community health systems remains uncertain. Therefore, we recommend that future research endeavors to evaluate the impact of such drastic health policy changes on the overall performance of CHS. Additionally, future studies should explore why the implementation period for the revised strategy (2019–2021) was short and whether it achieved the intended impact.

## Data Availability

The datasets used and/or analysed during the current study are available from the corresponding author on reasonable request.

## Abbreviations

CHWs: Community health workers
FGDs: Focus group discussions
FP/C: Family planning/contraceptive
HCPs: Healthcare providers
IDIs: In-depth interviews
NGO: Non-governmental organisation
NHC: Neighbourhood health committee
MoA: Ministry of Agriculture
WHO: World Health Organisation

## References

[1] Kutzin J, Sparkes SP. Health systems strengthening, universal health coverage, health security and resilience. Bull World Health Organ. 2016;94(1):2. 26769987 10.2471/BLT.15.165050 PMC4709803

[2] Sambo LG, Kirigia JM. Investing in health systems for universal health coverage in Africa. BMC Int Health Hum Rights. 2014;14(1):28. 25345988 10.1186/s12914-014-0028-5 PMC4422225

[3] Schneider H, Lehmann U. From community health workers to community health systems: time to widen the horizon? Health Syst Reform. 2016;2(2):112–8. 31514640 10.1080/23288604.2016.1166307

[4] Schneider H, et al. The multiple lenses on the Community Health System: implications for policy, practice and research. Int J Health Policy Manage. 2022;11(1):9. 10.34172/ijhpm.2021.73 PMC9278387 34273937

[5] Swider SM. Outcome effectiveness of community health workers: an integrative literature review. Public Health Nurs. 2002;19(1):11–20. 11841678 10.1046/j.1525-1446.2002.19003.x

[6] Mwai GW, et al. Role and outcomes of community health workers in HIV care in sub-saharan Africa: a systematic review. J Int AIDS Soc. 2013;16(1):18586. 24029015 10.7448/IAS.16.1.18586 PMC3772323

[7] World Health Organization. Strengthening health system governance: better policies, stronger performance. World Health Organization. Regional Office for Europe; 2016.

[8] George AS, Scott K, Mehra V, Sriram V. Synergies, strengths and challenges: findings on community capability from a systematic health systems research literature review. BMC Health Serv Res. 2016;16(7):623. 28185589 10.1186/s12913-016-1860-1 PMC5123247

[9] Alexander JA, et al. Challenges of capacity building in multisector community health alliances. Health Educ Behav. 2010;37(5):645–64. 20696883 10.1177/1090198110363883

[10] Tetui M, et al. Strengthening research and practice in community health systems: a research agenda and manifesto. Int J Health Policy Manage. 2022;11(1):17. 10.34172/ijhpm.2021.71 PMC9278394 34380193

[11] Jerome J, Ivers LC. Community health workers in health systems strengthening: a qualitative evaluation from rural Haiti. AIDS. 2010;24(Suppl 1):S67. 20023442 10.1097/01.aids.0000366084.75945.c9 PMC3169202

[12] Zambia M. National community health worker strategy in Zambia. Lusaka, Zambia: MOH Zambia; 2010.

[13] Zulu JM, Kinsman J, Michelo C, Hurtig A-K. Developing the national community health assistant strategy in Zambia: a policy analysis. Health Res Policy Syst. 2013;11(1):24. 23870454 10.1186/1478-4505-11-24 PMC3724745

[14] Shelley KD, et al. Implementation of the community health assistant (CHA) cadre in Zambia: a process evaluation to guide future scale-up decisions. J Community Health. 2016;41(2):398–408. 26547550 10.1007/s10900-015-0110-5

[15] Zambia M, Community Health S. 2017–2021 https://www.moh.gov.zm/docs/Community%20Health%20Strategy.pdf

[16] Zulu JM, et al. Exploring politics and contestation in the policy process: the case of Zambia’s contested community health strategy. Int J Health Policy Manage. 2022;11(1):24. 10.34172/ijhpm.2021.145 PMC9278392 34814675

[17] Simons H. Case study research: In-depth understanding in context. The Oxford handbook of qualitative research, 2014: pp. 455–470.

[18] Yin RK. Case Study Research: Design and Methods. Essential guide to qualitative methods in organizational research (Vol. 5). in The Information Systems Research Challenge (Harvard Business School Research Colloquium). London: Sage. 2009.

[19] Sarantakos S. Social research. Hampshire: Palgrave Macmillan; 2005. p. 464.

[20] Braun V, Clarke V. Using thematic analysis in psychology. Qualitative Res Psychol. 2006;3(2):77–101. 10.1191/1478088706qp063oa

[21] Milford C, et al. Teamwork in qualitative research: descriptions of a Multicountry Team Approach. Int J Qualitative Methods. 2017;16(1):1609406917727189. 10.1177/1609406917727189

[22] Association GA. World Medical Association Declaration of Helsinki: ethical principles for medical research involving human subjects. J Am Coll Dent. 2014;81(3):14–8. 25951678

[23] Zambia Ministry of National Planning. 2017–2021; https://planipolis.iiep.unesco.org/sites/default/files/ressources/zambia_national_development_plan_2017-2021.pdf

[24] Lane J, et al. Strengthening health policy development and management systems in low-and middle-income countries: South Africa’s approach. Health Policy OPEN. 2020;1:100010. 37383321 10.1016/j.hpopen.2020.100010 PMC10297791

[25] Sturmberg JP, Njoroge A. People-centred health systems, a bottom‐up approach: where theory meets empery. J Eval Clin Pract. 2017;23(2):467–73. 27062608 10.1111/jep.12540

[26] Buse K et al. Making Health Policy, 3e. 2023: McGraw Hill.

[27] Tummers L, Bekkers V. Policy implementation, street-level bureaucracy, and the importance of discretion. Public Manage Rev. 2014;16(4):527–47. 10.1080/14719037.2013.841978

[28] Hupe PL, Hill MJ. And the rest is implementation.’Comparing approaches to what happens in policy processes beyond great expectations. Public Policy Adm. 2016;31(2):103–21.

[29] Lehmann U, Gilson L. Actor interfaces and practices of power in a community health worker programme: a South African study of unintended policy outcomes. Health Policy Plann. 2013;28(4):358–66. 10.1093/heapol/czs066 22826517

[30] Lunsford SS, Fatta K, Stover KE, Shrestha R. Supporting close-to-community providers through a community health system approach: case examples from Ethiopia and Tanzania. Hum Resour Health. 2015;13(1):12. 25884699 10.1186/s12960-015-0006-6 PMC4387620

[31] Gram L, Daruwalla N, Osrin D. Understanding participation dilemmas in community mobilisation: can collective action theory help? J Epidemiol Community Health. 2019;73(1):90–6. 30377247 10.1136/jech-2018-211045 PMC6839791

[32] Sarriot E, Kouletio M. Community health systems as complex adaptive systems: ontology and praxis lessons from an urban health experience with demonstrated sustainability. Systemic Pract Action Res. 2015;28(3):255–72. 10.1007/s11213-014-9329-9

[33] Chavula MP, Zulu JM, Goicolea I, Hurtig A-K. Unlocking policy synergies, challenges and contradictions influencing implementation of the Comprehensive Sexuality Education Framework in Zambia: a policy analysis. Health Res Policy Syst. 2023;21(1):97. 37710251 10.1186/s12961-023-01037-y PMC10500755

[34] Silumbwe A, et al. The role of principled engagement in public health policymaking: the case of Zambia’s prolonged efforts to develop a comprehensive tobacco control policy. Global Health Action. 2023;16(1):2212959. 37212391 10.1080/16549716.2023.2212959 PMC10208213

[35] Zulu JM, Kinsman J, Michelo C, Hurtig A-K. Developing the national community health assistant strategy in Zambia: a policy analysis. Health Res Policy Syst. 2013;11:1–13. 23870454 10.1186/1478-4505-11-24 PMC3724745

[36] Doshmangir L, et al. Policy analysis of the Iranian Health Transformation Plan in primary healthcare. BMC Health Serv Res. 2019;19(1):670. 31533710 10.1186/s12913-019-4505-3 PMC6751681

[37] Assegaai T, Schneider H. The supervisory relationships of community health workers in primary health care: social network analysis of ward-based outreach teams in Ngaka Modiri Molema District, South Africa. BMJ Global Health, 2019. 4(6). 10.1136/bmjgh-2019-001839 PMC6936529 31908861

[38] Simooya C, et al. Exploring communication and implementation challenges of the HIV/AIDS policy change to test-and-treat-all in selected public health facilities in Lusaka District, Zambia. Implement Sci Commun. 2023;4(1):51. 37173757 10.1186/s43058-023-00430-6 PMC10176665

[39] Gilson L, Lehmann U, Schneider H. Practicing governance towards equity in health systems: LMIC perspectives and experience. Springer; 2017. 10.1186/s12939-017-0665-0 PMC5599870 28911320

[40] Walt G, Gilson L. Reforming the health sector in developing countries: the central role of policy analysis. Health Policy Plann. 1994;9(4):353–70. 10.1093/heapol/9.4.353 10139469

[41] Kanyamuna V, Mubita A, Kotzé DA. Is the policy environment for Zambia supportive of a thriving whole-ofgovernment monitoring and evaluation system. Adv Social Sci Res J. 2020;7(1):542–54.

[42] Shenton AK. Strategies for ensuring trustworthiness in qualitative research projects. Educ Inform. 2004;22(2):63–75. 10.3233/EFI-2004-22201

